# Supplementary material for: Synergistic Effect of a Flavonoid-Rich Cocoa–Carob Blend and Metformin in Preserving Pancreatic Beta Cells in Zucker Diabetic Fatty Rats
Source: Nutrients. 2024 Jan 17;16(2):273. doi: 10.3390/nu16020273 (PMC10821282; doi:10.3390/nu16020273)
Supplement: Supplementary file 1 [file nutrients-16-00273-s001.zip › nutrients-2764495-supplementary.pdf]

Supporting information:

Synergistic effect of a flavonoid-rich cocoa–carob blend and metformin in preserving pancreatic beta cells in Zucker diabetic fatty rats

Paula Gallardo-Villanueva, Tamara Fernández-Marcelo, Laura Villamayor, Angela M. Valverde, Sonia Ramos, Elisa Fernández-Millán, María Angeles Martín

***Supplementary Table S1.- Biochemical parameters** of Zucker lean rats (ZL); Zucker diabetic rats (ZDF); Zucker diabetic rats treated with metformin (ZDF (M)); Zucker diabetic rats fed with a CCB-rich diet (ZDF (CCB)), and Zucker diabetic rats treated with metformin and fed with a CCB-rich diet (ZDF (CCB+M)). Data represent the means ± SD of 6-8 animals. Means in a row without a common letter differ; $p < 0.05$.

	ZL	*ZDF*	*ZDF (M)*	*ZDF (CCB)*	*ZDF (CCB+M)*
Final body weight (g)	377 ± 17[a]	3930 ± 13[b]	423 ± 38[c]	384 ± 26[c]	411 ± 30[a]
Fasting glucose levels (mg/dL)	93.5 ± 14.3[a]	290.0 ± 32.7[b]	173.3 ± 16.9[c]	182.6 ± 19.0[c]	122.6 ± 16.0[a]
HbA1c (%)	4.8 ± 1.5[a]	19.3 ± 3.2[b]	5.3 ± 2.0[a]	14.3 ± 2.0[c]	4.6 ± 2.0[a]
Insulinemia (ng/dL)	0.45 ± 0.08[a]	0.37 ± 0.06[b]	0.53 ± 0.09[b]	0.51 ± 0.08[b]	0.49 ± 0.10[b]
HOMA-IR	2.2 ± 0.4[a]	8.7 ± 1.8[b]	4.8 ± 1.0[c]	4.4 ± 0.9[c]	5.2 ± 1.6[c]
HOMA-B	189.2 ± 14.8[a]	17.6 ± 5.1[b]	48.8 ± 13.2[c]	52.7 ± 13.3[c]	63.8 ± 12.3[d]

*Data obtained from *García-Diez et al, Antioxidants 2022, 11, 432*

“García-Díez, E.; López-Oliva, M.E.; Caro-Vadillo, A.; Pérez-Vizcaíno, F.; Pérez-Jiménez, J.; Ramos, S.; Martín, M.Á. Supplementation with a Cocoa–Carob Blend, Alone or in Combination with Metformin, Attenuates Diabetic Cardiomyopathy, Cardiac Oxidative Stress and Inflammation in Zucker Diabetic Rats. *Antioxidants* **2022**, 11, 432.
